# Supplementary material for: A viral protein promotes host SAMS1 activity and ethylene production for the benefit of virus infection
Source: eLife. 2017 Oct 10;6:e27529. doi: 10.7554/eLife.27529 (PMC5634785; doi:10.7554/eLife.27529)
Supplement: Supplementary file 1. [file elife-27529-supp1.docx]

**Supplementary file 1**

**Supplementary file 1A. Non-preference Test for Rice Varieties and Transgenic Lines Used in this Study, Related to Figure 1.**

| **Varieties** | **Non-preference^*1^** |
| --- | --- |
| WT | 1.90^a*2^ |
| *S11* OX#3 | 1.95^a^ |
| *S11* OX#5 | 1.93^a^ |
| *S11* OX#11 | 1.98^a^ |

*1, Non-preference was indicated by the number of leafhoppers settled on the individual plant.

*2, “a” means there is no significant difference (P value> 0.05) between these data.

**Supplementary file 1B. Non-preference Test for Rice Varieties and Transgenic Lines Used in this Study, Related to Figure4-figure supplement 1.**

| **Varieties** | **Non-preference** |
| --- | --- |
| WT | 1.95^a^ |
| *OsSAMS1* OX #10 | 2.05^a^ |
| *OsSAMS1* OX #17 | 1.97^a^ |
| *OsSAMS1* OX #25 | 1.98^a^ |
| *OsSAMS1* RNAi-S | 1.85^a^ |
| *OsSAMS1* RNAi-W | 2.20^a^ |

**Supplementary file 1C. Non-preference Test for Rice Varieties and Transgenic Lines Used in this Study, Related to Figure 4.**

| **Varieties** | **Non-preference** |
| --- | --- |
| WT | 2.01^a^ |
| *OsSAMS1* OX #10 | 1.87^a^ |
| *OsSAMS1* OX #17 | 1.93^a^ |
| *OsSAMS1* OX #25 | 2.00^a^ |
| *Ossams1* KO #31 | 1.91^a^ |
| *Ossams1* KO #39 | 2.05^a^ |

**Supplementary file 1D. Non-preference Test for Rice Varieties and Transgenic Lines Used in this Study, Related to Figure 5.**

| **Varieties** | **Non-preference** |
| --- | --- |
| WT | 1.95^a^ |
| *OsEIN2* OX #2 | 1.97^a^ |
| *OsEIN2* OX #3 | 2.03^a^ |
| *osein2* | 2.05^a^ |

**Supplementary file 1E. Non-preference Test for Rice Varieties and Transgenic Lines Used in this Study, Related to Figure 5-figure supplement 2.**

| **Varieties** | **Non-preference** |
| --- | --- |
| WT | 1.95^a^ |
| *osein2* | 2.03^a^ |
| J119#1 | 2.05^a^ |
| J119#2 | 1.98^a^ |
| J119#3 | 2.03^a^ |

**Supplementary file 1F. Non-preference Test for WT Rice Plants with Indicated Treatment, Related to Figure 6.**

| **Varieties** | **Non-preference** |
| --- | --- |
| H_2_O | 2.03 ^a^ |
| ACC | 1.98^a^ |
| AVG | 1.98^a^ |
