# Supplementary material for: A viral protein promotes host SAMS1 activity and ethylene production for the benefit of virus infection
Source: eLife. 2017 Oct 10;6:e27529. doi: 10.7554/eLife.27529 (PMC5634785; doi:10.7554/eLife.27529)
Supplement: Supplementary file 2. [file elife-27529-supp2.docx]

**Supplementary file 2**

**Supplementary file 2A. Record of the Number of Rice Plants Showing RDV Symptoms at Time Course (Week Post Inoculation, WPI) for WT, *S11* OX#3, *S11* OX#5, *S11* OX#11 Related to Figure 1 and Experimental Procedures**

|  |  | WT  (Total: 30/repeat) **^*1^** | *S11* OX#3  (Total: 30/repeat) | *S11* OX#5 (Total: 30/repeat) | *S11* OX#11 (Total: 30/repeat) |
| --- | --- | --- | --- | --- | --- |
| 1wpi | R1^*2^ | 0 | 0 | 0 | 2 |
|  | R2 | 0 | 0 | 0 | 2 |
|  | R3 | 0 | 1 | 2 | 2 |
| 2wpi | R1 | 5 | 5 | 8 | 10 |
|  | R2 | 3 | 5 | 7 | 8 |
|  | R3 | 7 | 5 | 7 | 10 |
| 3wpi | R1 | 10 | 12 | 12 | 13 |
|  | R2 | 8 | 10 | 8 | 12 |
|  | R3 | 12 | 10 | 13 | 17 |
| 4wpi | R1 | 17 | 17 | 18 | 18 |
|  | R2 | 14 | 17 | 17 | 20 |
|  | R3 | 16 | 17 | 20 | 23 |
| 5wpi | R1 | 18 | 18 | 25 | 22 |
|  | R2 | 20 | 18 | 22 | 27 |
|  | R3 | 22 | 23 | 22 | 25 |
| 6wpi | R1 | 22 | 23 | 25 | 24 |
|  | R2 | 23 | 23 | 25 | 27 |
|  | R3 | 22 | 23 | 23 | 27 |
| 7wpi | R1 | 23 | 25 | 26 | 26 |
|  | R2 | 25 | 23 | 27 | 28 |
|  | R3 | 23 | 23 | 25 | 27 |
| 8wpi | R1 | 25 | 25 | 26 | 27 |
|  | R2 | 28 | 25 | 28 | 28 |
|  | R3 | 25 | 25 | 27 | 28 |

*1: For each repeat, 30 seedlings were inoculated with viruliferous leafhopper.

*2: R1 means biological Repeat 1.

**Statistical analysis of infection rate for WT, *S11* OX#3, *S11* OX#5, *S11* OX#11 Related to Figure 1 and Experimental Procedures**

| P value | | *S11* OX#3 | *S11* OX#5 | *S11* OX#11 |
| --- | --- | --- | --- | --- |
| Relative to WT | 1wpi | — | — | — |
|  | 2wpi | — | — | * |
|  | 3wpi | — | — | — |
|  | 4wpi | — | — | — |
|  | 5wpi | — | — | — |
|  | 6wpi | — | — | * |
|  | 7wpi | — | — | * |
|  | 8wpi | — | — | — |

“—” represents no significant difference, **P*<0.05.

**Supplementary file 2B. Record of the Number of Rice Plants Showing RDV Symptoms at Time Course (Week Post Inoculation, WPI) for WT, *OsSAMS1* OX#10, OX#17, OX#25, RNAi-W, RNAi-S, Related to Figure4-figure supplement 1 and Experimental Procedures**

|  |  | WT  (Total: 30/repeat) | *OsSAMS1* OX#10  (Total: 30/repeat) | *OsSAMS1* OX#17  (Total:  30/repeat) | *OsSAMS1* OX#25  (Total:  30/repeat) | *OsSAMS1* RNAi-W(Total:  30/repeat) | *OsSAMS1* RNAi-S(Total:  30/repeat) |
| --- | --- | --- | --- | --- | --- | --- | --- |
| 1wpi | R1 | 0 | 2 | 2 | 0 | 0 | 0 |
|  | R2 | 2 | 0 | 2 | 2 | 0 | 0 |
|  | R3 | 0 | 0 | 2 | 0 | 0 | 0 |
| 2wpi | R1 | 6 | 8 | 9 | 6 | 2 | 0 |
|  | R2 | 6 | 8 | 9 | 8 | 3 | 3 |
|  | R3 | 8 | 5 | 9 | 9 | 2 | 2 |
| 3wpi | R1 | 12 | 15 | 17 | 15 | 8 | 6 |
|  | R2 | 12 | 15 | 17 | 15 | 8 | 6 |
|  | R3 | 12 | 11 | 12 | 11 | 5 | 5 |
| 4wpi | R1 | 15 | 18 | 23 | 21 | 11 | 12 |
|  | R2 | 17 | 20 | 24 | 23 | 12 | 12 |
|  | R3 | 15 | 17 | 18 | 17 | 6 | 8 |
| 5wpi | R1 | 18 | 21 | 26 | 17 | 15 | 14 |
|  | R2 | 18 | 23 | 27 | 26 | 17 | 15 |
|  | R3 | 18 | 20 | 20 | 21 | 12 | 11 |
| 6wpi | R1 | 24 | 21 | 26 | 24 | 18 | 15 |
|  | R2 | 23 | 24 | 27 | 26 | 20 | 15 |
|  | R3 | 24 | 24 | 26 | 24 | 17 | 15 |
| 7wpi | R1 | 27 | 24 | 29 | 24 | 21 | 20 |
|  | R2 | 24 | 26 | 30 | 27 | 23 | 20 |
|  | R3 | 24 | 26 | 29 | 26 | 21 | 20 |
| 8wpi | R1 | 27 | 24 | 29 | 27 | 21 | 21 |
|  | R2 | 27 | 27 | 30 | 29 | 23 | 20 |
|  | R3 | 26 | 27 | 30 | 29 | 21 | 21 |

**Statistical analysis of infection rate for WT, *OsSAMS1* OX#10, OX#17, OX#25, RNAi-W, RNAi-S, Related to Figure4-figure supplement 1 and Experimental Procedures**

| P value | | *OsSAMS1* OX#10 | *OsSAMS1* OX#17 | *OsSAMS1* OX#25 | *OsSAMS1* RNAi-W | *OsSAMS1* RNAi-S |
| --- | --- | --- | --- | --- | --- | --- |
| Relative to WT | 1wpi | — | — | — | — | — |
|  | 2wpi | — | * | — | ** | * |
|  | 3wpi | — | — | — | ** | ** |
|  | 4wpi | — | * | * | * | * |
|  | 5wpi | * | * | — | — | * |
|  | 6wpi | — | ** | — | ** | ** |
|  | 7wpi | — | * | — | — | ** |
|  | 8wpi | — | ** | — | ** | ** |

“—” represents no significant difference, **P*<0.05, ***P*<0.01.

**Supplementary file 2C. Record of the Number of Rice Plants Showing RDV Symptoms at Time Course (Week Post Inoculation, WPI) for WT, *OsSAMS1* OX#10, OX#17, OX#25, KO#31, KO#39, Related to Figure 4 and Experimental Procedures**

|  |  | WT  (Total: 30/repeat) | *OsSAMS1* OX#10  (Total: 30/repeat) | *OsSAMS1* OX#17  (Total:  30/repeat) | *OsSAMS1* OX#25  (Total:  30/repeat) | *Ossams1* KO#31  (Total:  30/repeat  ) | *Ossams1* KO#39  (Total:  30/repeat  ) |
| --- | --- | --- | --- | --- | --- | --- | --- |
| 1wpi | R1 | 0 | 2 | 1 | 2 | 0 | 0 |
|  | R2 | 0 | 1 | 0 | 1 | 0 | 0 |
|  | R3 | 2 | 0 | 2 | 1 | 0 | 0 |
| 2wpi | R1 | 9 | 11 | 11 | 12 | 3 | 5 |
|  | R2 | 6 | 14 | 12 | 14 | 5 | 5 |
|  | R3 | 8 | 12 | 12 | 11 | 3 | 0 |
| 3wpi | R1 | 14 | 17 | 18 | 17 | 11 | 9 |
|  | R2 | 14 | 17 | 18 | 17 | 8 | 5 |
|  | R3 | 14 | 18 | 15 | 15 | 5 | 3 |
| 4wpi | R1 | 17 | 18 | 23 | 23 | 12 | 9 |
|  | R2 | 16 | 20 | 24 | 23 | 11 | 6 |
|  | R3 | 18 | 20 | 23 | 21 | 8 | 5 |
| 5wpi | R1 | 18 | 20 | 24 | 24 | 17 | 14 |
|  | R2 | 18 | 21 | 24 | 24 | 14 | 12 |
|  | R3 | 23 | 20 | 24 | 24 | 15 | 12 |
| 6wpi | R1 | 23 | 21 | 24 | 24 | 20 | 17 |
|  | R2 | 21 | 26 | 25 | 24 | 14 | 20 |
|  | R3 | 24 | 24 | 24 | 25 | 18 | 17 |
| 7wpi | R1 | 26 | 24 | 26 | 26 | 20 | 18 |
|  | R2 | 24 | 26 | 26 | 24 | 20 | 21 |
|  | R3 | 26 | 26 | 24 | 26 | 21 | 21 |
| 8wpi | R1 | 26 | 26 | 27 | 26 | 21 | 20 |
|  | R2 | 24 | 26 | 26 | 27 | 20 | 23 |
|  | R3 | 26 | 27 | 24 | 26 | 23 | 23 |

**Statistical analysis of infection rate for WT, *OsSAMS1* OX#10, OX#17, OX#25, KO#31, KO#39, Related to Figure 4 and Experimental Procedures**

| P value | | *OsSAMS1* OX#10 | *OsSAMS1* OX#17 | *OsSAMS1* OX#25 | *Ossams1* KO#31 | *Ossams1* KO#39 |
| --- | --- | --- | --- | --- | --- | --- |
| Relative to WT | 1wpi | — | — | — | — | — |
|  | 2wpi | * | * | * | * | — |
|  | 3wpi | ** | * | * | * | ** |
|  | 4wpi | — | ** | ** | ** | ** |
|  | 5wpi | — | — | — | — | * |
|  | 6wpi | — | — | — | * | * |
|  | 7wpi | — | — | — | ** | * |
|  | 8wpi | — | — | — | * | — |

“—” represents no significant difference, **P*<0.05, ***P*<0.01.

**Supplementary file 2D. Record of the Number of Rice Plants Showing RDV Symptoms at Time Course (Week Post Inoculation, WPI) for WT, *OsEIN2* OX#2, OX#3, *osein2*, Related to Figure 5 and Experimental Procedures**

|  |  | WT  (Total: 30/repeat) | *OsEIN2* OX#2  (Total: 30/repeat) | *OsEIN2* OX#3  (Total: 30/repeat) | *osein2*  (Total: 30/repeat) |
| --- | --- | --- | --- | --- | --- |
| 1wpi | R1 | 0 | 3 | 1 | 0 |
|  | R2 | 0 | 0 | 0 | 0 |
|  | R3 | 0 | 0 | 0 | 0 |
| 2wpi | R1 | 8 | 12 | 12 | 0 |
|  | R2 | 6 | 14 | 15 | 0 |
|  | R3 | 8 | 11 | 14 | 0 |
| 3wpi | R1 | 12 | 18 | 20 | 5 |
|  | R2 | 11 | 15 | 17 | 0 |
|  | R3 | 11 | 18 | 18 | 2 |
| 4wpi | R1 | 18 | 20 | 24 | 9 |
|  | R2 | 15 | 20 | 21 | 3 |
|  | R3 | 14 | 20 | 23 | 2 |
| 5wpi | R1 | 20 | 26 | 26 | 14 |
|  | R2 | 17 | 24 | 23 | 5 |
|  | R3 | 18 | 26 | 26 | 5 |
| 6wpi | R1 | 23 | 26 | 29 | 14 |
|  | R2 | 20 | 26 | 27 | 8 |
|  | R3 | 20 | 27 | 30 | 9 |
| 7wpi | R1 | 26 | 27 | 30 | 14 |
|  | R2 | 24 | 30 | 27 | 10 |
|  | R3 | 24 | 27 | 30 | 14 |
| 8wpi | R1 | 26 | 29 | 30 | 14 |
|  | R2 | 26 | 30 | 29 | 13 |
|  | R3 | 24 | 27 | 30 | 14 |

**Statistical analysis of infection rate for WT, *OsEIN2* OX#2, OX#3, *osein2*, Related to Figure 5 and Experimental Procedures**

| P value | | *OsEIN2* OX#2 | *OsEIN2* OX#3 | *osein2* |
| --- | --- | --- | --- | --- |
| Relative to WT | 1wpi | — | — | — |
|  | 2wpi | * | ** | ** |
|  | 3wpi | ** | ** | ** |
|  | 4wpi | * | ** | * |
|  | 5wpi | ** | ** | * |
|  | 6wpi | ** | ** | ** |
|  | 7wpi | — | * | ** |
|  | 8wpi | * | ** | ** |

“—” represents no significant difference, **P*<0.05, ***P*<0.01.

**Supplementary file 2E. Record of the Number of Rice Plants Showing RDV Symptoms at Time Course (Week Post Inoculation, WPI) for WT, *osein2*, J119#1, J119#2, J119#3, Related to Figure 5-figure supplement 2 and Experimental Procedures**

|  |  | WT  (Total: 30/repeat) | *osein2*  (Total: 30/repeat) | J119#1  (Total: 30/repeat) | J119#2 (Total: 30/repeat) | J119#3 (Total: 30/repeat) |
| --- | --- | --- | --- | --- | --- | --- |
| 1wpi | R1 | 2 | 0 | 0 | 0 | 0 |
|  | R2 | 0 | 0 | 0 | 0 | 0 |
|  | R3 | 2 | 0 | 0 | 0 | 0 |
| 2wpi | R1 | 5 | 0 | 0 | 0 | 2 |
|  | R2 | 2 | 0 | 0 | 0 | 2 |
|  | R3 | 6 | 0 | 0 | 2 | 0 |
| 3wpi | R1 | 9 | 3 | 5 | 5 | 3 |
|  | R2 | 5 | 2 | 2 | 3 | 2 |
|  | R3 | 9 | 2 | 2 | 3 | 3 |
| 4wpi | R1 | 14 | 6 | 11 | 9 | 8 |
|  | R2 | 11 | 3 | 6 | 6 | 5 |
|  | R3 | 14 | 3 | 3 | 6 | 6 |
| 5wpi | R1 | 18 | 8 | 12 | 11 | 9 |
|  | R2 | 18 | 6 | 11 | 8 | 8 |
|  | R3 | 21 | 6 | 6 | 8 | 8 |
| 6wpi | R1 | 21 | 12 | 14 | 13 | 11 |
|  | R2 | 23 | 11 | 14 | 9 | 11 |
|  | R3 | 23 | 8 | 9 | 9 | 9 |
| 7wpi | R1 | 23 | 14 | 14 | 14 | 11 |
|  | R2 | 23 | 12 | 15 | 11 | 12 |
|  | R3 | 24 | 10 | 9 | 9 | 11 |
| 8wpi | R1 | 23 | 15 | 14 | 14 | 14 |
|  | R2 | 24 | 14 | 15 | 14 | 15 |
|  | R3 | 24 | 13 | 15 | 12 | 12 |

**Statistical analysis of infection rate for WT, *osein2*, J119#1, J119#2, J119#3, Related to Figure 5-figure supplement 2 and Experimental Procedures**

| P value | | *osein2* | J119#1 | J119#2 | J119#3 |
| --- | --- | --- | --- | --- | --- |
| Relative to WT | 1wpi | — | — | — | — |
|  | 2wpi | * | * | — | — |
|  | 3wpi | * | * | — | * |
|  | 4wpi | ** | — | * | ** |
|  | 5wpi | ** | * | ** | ** |
|  | 6wpi | ** | ** | ** | ** |
|  | 7wpi | ** | ** | ** | ** |
|  | 8wpi | ** | ** | ** | ** |

“—” represents no significant difference, **P*<0.05, ***P*<0.01.

**Supplementary file 2F. Record of the Number of Rice Plants Showing RDV Symptoms at Time Course (Week Post Inoculation, WPI) for Rice Pretreated with H_2_O, ACC and AVG with Root Immersing Method, Related to Figure 6 and Experimental Procedures**

|  |  | H_2_O  (Total: 30/repeat) | ACC  (Total: 30/repeat) | AVG  (Total: 30/repeat) |
| --- | --- | --- | --- | --- |
| 1wpi | R1 | 0 | 0 | 0 |
|  | R2 | 0 | 2 | 0 |
|  | R3 | 0 | 0 | 0 |
| 2wpi | R1 | 3 | 5 | 2 |
|  | R2 | 3 | 7 | 3 |
|  | R3 | 5 | 8 | 2 |
| 3wpi | R1 | 7 | 12 | 3 |
|  | R2 | 7 | 10 | 3 |
|  | R3 | 8 | 13 | 5 |
| 4wpi | R1 | 12 | 18 | 7 |
|  | R2 | 10 | 15 | 5 |
|  | R3 | 12 | 18 | 7 |
| 5wpi | R1 | 18 | 25 | 12 |
|  | R2 | 17 | 27 | 8 |
|  | R3 | 15 | 23 | 8 |
| 6wpi | R1 | 23 | 28 | 15 |
|  | R2 | 20 | 27 | 13 |
|  | R3 | 20 | 28 | 12 |
| 7wpi | R1 | 27 | 29 | 22 |
|  | R2 | 23 | 28 | 20 |
|  | R3 | 25 | 30 | 18 |
| 8wpi | R1 | 27 | 29 | 22 |
|  | R2 | 26 | 30 | 23 |
|  | R3 | 28 | 30 | 20 |

**Statistical analysis of infection rate for Rice Pretreated with H_2_O, ACC and AVG with Root Immersing Method, Related to Figure 6 and Experimental Procedures**

| P value | | ACC | AVG |
| --- | --- | --- | --- |
| Relative to H_2_O | 1wpi | — | — |
|  | 2wpi | — | — |
|  | 3wpi | * | ** |
|  | 4wpi | ** | ** |
|  | 5wpi | ** | * |
|  | 6wpi | ** | ** |
|  | 7wpi | * | * |
|  | 8wpi | * | ** |

“—” represents no significant difference, **P*<0.05, ***P*<0.01.
