# Supplementary material for: A viral protein promotes host SAMS1 activity and ethylene production for the benefit of virus infection
Source: eLife. 2017 Oct 10;6:e27529. doi: 10.7554/eLife.27529 (PMC5634785; doi:10.7554/eLife.27529)
Supplement: Supplementary file 5. — (A) Constructs in this study. (B) Primers for plasmids constructions in this study. (C) LC-MS/MS conditions. (D) Primers for RNA gel blot probes, real-time PCR, and semi-quantitative RT-PCR. [file elife-27529-supp5.docx]

**Supplementary file 5**

**Supplementary file 5A. Constructs, Related to Experimental Procedures**

| **use** | **Construct** | **Plasmid name** | **Insert Fragment** | **Primers** | **Template** | **Plasmid backbone** | **Cloning method** |
| --- | --- | --- | --- | --- | --- | --- | --- |
| Yeast  Two-  hybrid | Bait-S11 | pDHB1-S11 | S11 | S11F1  S11R1 | RDV-infected rice cDNA | pDHB1 | *Sfi* I  *Sfi* I |
|  | Prey-OsSAMS1 | pPR3-N-OsSAMS1 | OsSAMS1 | OsSAMS1F1  OsSAMS1R1 | Rice cDNA | pPR3-N | *Sfi* I  *Sfi* I |
|  | Prey-OsSAMS2 | pPR3-N-OsSAMS2 | OsSAMS2 | OsSAMS2F1  OsSAMS2R1 | Rice cDNA | pPR3-N | *Sfi* I  *Sfi* I |
|  | Prey-OsSAMS3 | pPR3-N-OsSAMS3 | OsSAMS3 | OsSAMS3F1  OsSAMS3R1 | Rice cDNA | pPR3-N | *Sfi* I  *Sfi* I |
| Transgene rice and transient expression in tobacco | 35S:FLAG-  OsSAMS1 | p2300-FLAG-OsSAMS1 | FLAG-OsSAMS1 | OsSAMS1F2  OsSAMS1R2 | pPR3-N-OsSAMS1 | pCAMBIA2300 | *Sma* I  *Xba* I |
|  | 35S:FLAG-  OsSAMS2 | p2300-FLAG-OsSAMS2 | FLAG-OsSAMS2 | OsSAMS2F2  OsSAMS2R2 | pPR3-N-OsSAMS2 | pCAMBIA2300 | *Xba* I  *Pst* I |
|  | 35S:FLAG-  OsSAMS3 | P2300-FLAG-OsSAMS3 | FLAG-OsSAMS3 | OsSAMS3F2  OsSAMS3R2 | pPR3-N-OsSAMS3 | pCAMBIA2300 | *Sma* I  *Bam*H I |
|  | 35S:HA-Pns11 | pWM101-HA-S11 | HA-S11 | S11F2  S11R2 | pDHB1-S11 | pWM101 | *Kpn* I  *Sal* I |
|  | Ubi:FLAG-OsSAMS1 | p2300-Ubi-FLAG-OsSAMS1 | FLAG-OsSAMS1 | OsSAMS1F3  OsSAMS1R3 | pPR3-N-OsSAMS1 | pCAMBIA2300 | *Kpn* I  *Sma* I |
|  | Actin:  OsSAMS1RNAi | p2300-Act-OsSAMS1RNAi | OsSAMS1 and OsSAMS1IR | OsSAMS1F4  OsSAMS1R4 | pPR3-N-OsSAMS1 | pCAMBIA2300 | *Bgl* II  *Xho* I |
|  | Ubi:HA-Pns11 | p2300-Ubi-HA-S11 | HA-S11 | S11F2  S11R3 | pDHB1-S11 | pCAMBIA2300 | *Kpn* I  *Sma* I |
| LCI assay | cLUC-OsSAMS1 | p1300-cLUC-OsSAMS1 | OsSAMS1 | OsSAMS1F3  OsSAMS1R5 | pPR3-N-OsSAMS1 | pCMABIA1300 | *Kpn* I  *Kpn* I |
|  | S11-nLUC | p1300-nLUC-S11 | S11 | S11F2  S11R4 | pDHB1-S11 | pCMABIA1300 | *Kpn* I  *Sal* I |
| Protein puri  -fication | MBP-Pns11 | pMAL-p2x- Pns11 | S11 | S11F3  S11R5 | pDHB1-S11 | pMAL-p2x | *Sal* I  *Pst* I |
|  | MBP-P9 | pMAL-p2x- P9 | S9 | S9F1  S9R1 | pGAD-S9 | pMAL-p2x | *Bam*H I  *Sal* I |
|  | MBP-GFP | pMAL-p2x- GFP | GFP | GFPF1  GFPR1 | pRTL2-GFP | pMAL-p2x | *Bam*H I  *Sal* I |
|  | GST-OsSAMS1 | pGEX-OsSAMS1 | OsSAMS1 | OsSAMS1F3  OsSAMS1R6 | pPR3-N-OsSAMS1 | pGEX-4T-1 | *Kpn* I  *Pst* I |
|  | GST-OsSAMS2 | pGEX-OsSAMS2 | OsSAMS2 | OsSAMS2F3  OsSAMS2R2 | pPR3-N-OsSAMS2 | pGEX-4T-1 | *Kpn* I  *Pst* I |
| BiFC | OsSAMS1-YFP^N^ | p2YN-OsSAMS1 | OsSAMS1 | OsSAMS1F5  OsSAMS1R7 | pPR3-N-OsSAMS1 | p2YN | *Pac* I  *Spe* I |
|  | OsSAMS1-YFP^C^ | p2YC-OsSAMS1 | OsSAMS1 | OsSAMS1F5  OsSAMS1R7 | pPR3-N-OsSAMS1 | p2YC | *Pac* I  *Spe* I |
|  | Pns11-YFP^N^ | p2YN-Pns11 | S11 | S11F4  S11R6 | pDHB1-S11 | p2YN | *Pac* I  *Spe* I |
|  | Pns11-YFP^C^ | p2YC-Pns11 | S11 | S11F4  S11R6 | pDHB1-S11 | p2YC | *Pac* I  *Spe* I |

**Supplementary file 5B. Primers for Plasmids Constructions, Related to Experimental Procedures**

| **Primer name** | **Primer Sequence 5’-3’** |
| --- | --- |
| S11F1 | GGCCATTACGGCCATGAGTGGAACATTACCCTTGGCTATGAC |
| S11R1 | GGCCGAGGCGGCCTTACTTACGCTTTGATTTGCGAGTATTGG |
| S11F2 | GGTACCATGAGTGGAACATTACCCTTGGCTATGAC |
| S11R2 | GTCGACTTACTTACGCTTTGATTTGCGAGTATTGG |
| S11R3 | CCCGGGTTACGCTTTGATTTGCGAGTATTGG |
| S11R4 | GTCGACCTTACGCTTTGATTTGCGAGTATTGG |
| S11F3 | GTCGACAGTGGAACATTACCCTTGGCTATGAC |
| S11R5 | CTGCAGTTACTTACGCTTTGATTTGCGAGTATTGG |
| S11F4 | TTAATTAAATGAGTGGAACATTACCCTTGGCTATGAC |
| S11R6 | ACTAGTCTTACGCTTTGATTTGCGAGTATTGG |
| S9F1 | GGATCCATGGGTAAGCTCCAAGATGGAATCG |
| S9R1 | GTCGACTCAAACTGAGGGTGCGAGTCCTAAC |
| GFPF1 | GGATCCATGGTGAGCAAGGGCGAGGAG |
| GFPR1 | GTCGACTTACTTGTACAGCTCGTCCATGCCG |
| OsSAMS1F1 | GGCCATTACGGCCATGGCCGCACTTGATACCTTCCTC |
| OsSAMS1R1 | GGCCGAGGCGGCCTTAGGCAGAAGGCTTCTCCCACTTG |
| OsSAMS1F2 | CCCGGGATGGCCGCACTTGATACCTTCCTC |
| OsSAMS1R2 | TCTAGATTAGGCAGAAGGCTTCTCCCACTTG |
| OsSAMS1F3 | GGTACCATGGCCGCACTTGATACCTTCCTC |
| OsSAMS1R3 | CCCGGGTTAGGCAGAAGGCTTCTCCCACTTG |
| OsSAMS1F4 | CTCGAGAAGCTCCCTTTCGGAGGCTTTTGC |
| OsSAMS1F5 | TTAATTAAATGGCCGCACTTGATACCTTCCTC |
| OsSAMS1R4 | AGATCTATTTCTTCTTCTCAAAGTAGTGTAGCATCTTTC |
| OsSAMS1R5 | GGTACCTTAGGCAGAAGGCTTCTCCCACTTG |
| OsSAMS1R6 | CTGCAGTTAGGCAGAAGGCTTCTCCCACTTG |
| OsSAMS1R7 | ACTAGTGGCAGAAGGCTTCTCCCACTTG |
| OsSAMS2F1 | GGCCATTACGGCCATGGCGGCGGAGACGTTCCT |
| OsSAMS2R1 | GGCCGAGGCGGCCTCAGGAAGATGCCTTCTCATACTTGAG |
| OsSAMS2F2 | TCTAGAATGGCGGCGGAGACGTTCCT |
| OsSAMS2R2 | CTGCAGTCAGGAAGATGCCTTCTCATACTTGAG |
| OsSAMS2F3 | GGTACCATGGCGGCGGAGACGTTCCT |
| OsSAMS3F1 | GGCCATTACGGCCATGGCTGAGGTTGACACCTTCCTCTTC |
| OsSAMS3R1 | GGCCGAGGCGGCCTTATGCAGAAGGCTCCTCCCACTTG |
| OsSAMS3F2 | CCCGGGATGGCTGAGGTTGACACCTTCCTCTTC |
| OsSAMS3R2 | GGATCCTTATGCAGAAGGCTCCTCCCACTTG |

**Supplementary file 5C. LC-MS/MS conditions**

| **Column** | Agilent Zorbax Eclipse Plus C18, 2.1*50mm, 1.8-Micron | | |
| --- | --- | --- | --- |
| **Column Temperature** | 25℃ | | |
| **Buffer A** | 5 mM Ammonium Acetate + 0.1% Acetic Acid | | |
| **Buffer B** | Acetonitrile | | |
| **Time** | **A%** | **B%** | **Flow (ml/min)** |
| 0 | 98 | 2 | 0.3 |
| 1 | 98 | 2 |  |
| 2 | 85 | 15 |  |
| 3 | 40 | 60 |  |
| 4 | 40 | 60 |  |
| 4.5 | 98 | 2 |  |
| 7 | 98 | 2 |  |

| **Compound Name** | **Precursor Ion** | **Product Ion** | **Polarity** |
| --- | --- | --- | --- |
| SAM | 399 | 249.5 | Positive |
| SAM | 399 | 135.6 | Positive |

**Supplementary file 8D. Primers for RNA Gel Blot Probes, Real-time PCR, and Semi-Quantitative RT-PCR, Related to Experimental Procedures**

| **Primer name** | **Primer Sequence 5’-3’** | **Use** |
| --- | --- | --- |
| *EF1α-F* | GCACGCTCTTCTTGCTTTCACTCT | Real-Time PCR |
| *EF1α-R* | GCACGCTCTTCTTGCTTTCACTCT |  |
| *OsERF3-F* | CACACCCAAACCCAACCTCCC |  |
| *OsERF3-R* | CCCGGAACTCTCGAGCGG |  |
| *OsPR1a-F* | CGTCTTCATCACCTGCAACTACTC |  |
| *OsPR1a-R* | CATGCATAAACACGTAGCATAGCA |  |
| *OsPR1b-F* | GGCAACTTCGTCGGACAGA |  |
| *OsPR1b-R* | CCGTGGACCTGTTTACATTTTCA |  |
| *OsPR2-F* | TTCCGTTTTAACACTGGCATTG |  |
| *OsPR2-R* | TAGCACACGCCGATAGATTG |  |
| *OsPR3-F* | GAGATCGCTGCCTTCTTC |  |
| *OsPR3-R* | GCTCTTGTCACAATAGTCCAT |  |
| *OsPR5-F* | CAACAGCAACTACCAAGTCGTCTT |  |
| *OsPR5-R* | CAAGGTGTCGTTTTATTCATCAACTTT |  |
| *OsPR10-F* | CCCTGCCGAATACGCCTAA |  |
| *OsPR10-R* | CTCAAACGCCACGAGAATTTG |  |
| *OsNH1-F* | CACGCCTAAGCCTCGGATTA |  |
| *OsNH1-R* | TCAGTGAGCAGCATCCTGACTAG |  |
| *OsCht1-F* | CGTGGTGACCAACATCATCA |  |
| *OsCht1-R* | GAGTTGAAAGGCCTCTGGTTGT |  |
| *OsWRKY13-F* | TTTGGGAAAGCGTTGATTAGT |  |
| *OsWRKY13-R* | GCGCACACACACTCCAACTC |  |
| *OsMPK6-F* | TTGCTACGAGGGCTAAAATATGTG |  |
| *OsMPK6-R* | GGAACAAATTGCTTGGCTTCA |  |
| *OsERF1-F* | GACCTCGGAGTCGTCCTTCT |  |
| *OsERF1-R* | TCTCTTTCTCCGTTTCGGG |  |
| *S2-F* | AACTTTGCTTCGGTGGTTGCCCCTG | RT-PCR and Probe Synthesis |
| *S2-R* | GCTATACACATCATCGCCGTGGTGT |  |
| *S8-F* | GATATACCCTATTCTGAACCTATTG |  |
| *S8-R* | GTTATCGAGTTCAATGTATAGTAGG |  |
| *S11-F* | ATGAGTGGAACATTACCCTTGG |  |
| *S11-R* | TTACTTACGCTTTGATTTGCG |  |
| *OsSAMS1-F* | ACTTGATACCTTCCTCTTTACCTCGG |  |
| *OsSAMS1-R* | TCAGGCCTGAGCCATGCGC |  |
