# Supplementary material for: A viral protein promotes host SAMS1 activity and ethylene production for the benefit of virus infection
Source: eLife. 2017 Oct 10;6:e27529. doi: 10.7554/eLife.27529 (PMC5634785; doi:10.7554/eLife.27529)
Supplement: Transparent reporting form [file elife-27529-transrepform.docx]

***eLife’s* transparent reporting form**

We encourage authors to provide detailed information *within their submission* to facilitate the interpretation and replication of experiments. If you have any questions, please contact us: [editorial@elifesciences.org](mailto:editorial@elifesciences.org).

**Sample-size estimation**

- You should state whether an appropriate sample size was computed when the study was being designed
- You should state the statistical method of sample size computation and any required assumptions
- If no explicit power analysis was used, you should describe how you decided what sample (replicate) size (number) to use

Please outline where this information can be found within the submission (e.g., page numbers or figure legends), or explain why this information doesn’t apply to your submission:

n=6 rice plants from 30 rice plants used for northern and western blot assays, see Figure 1B and C, 4B and C, 5B and C, 6G and H, Figure 4-figure supplement 1B and C, Figure 5-figure supplement 2B and C

30 rice plants used for virus-infection assay each and analysis of infection rate, see Figure 1D, 4D, 5D, 6I, Figure 4-figure supplement 1D and Figure 5-figure supplement 2D (details see Supplementary file 2).

n=6 rice plants from each rice line for SAM, ACC and ethylene measurements, see Figure 3, Figure 3-figure supplement 2, Figure 6B-D and materials and methods. Tukey’s honestly significant difference post hoc test was performed for multiple comparison.

n=6 rice plants from each rice line for gene expression analysis, see Figure 6E, Figure 2-figure supplement 1D and E, Figure 3-figure supplement 1A-C, and Figure 5-figure supplement 3.

n=100 from 8 rice plants for analysis of seed length and width, see Figure 1-figure supplement 1C and D and Figure 5-figure supplement 1D and E . Tukey’s honestly significant difference post hoc test was performed for multiple comparison.

**Replicates**

- You should report how often each experiment was performed
- You should include a definition of biological versus technical replication
- The data obtained should be provided and sufficient information should be provided to indicate the number of independent biological and/or technical replicates
- If you encountered any outliers, you should describe how these were handled
- Criteria for exclusion/inclusion of data should be clearly stated
- High-throughput sequence data should be uploaded before submission, with a private link for reviewers provided (these are available from both GEO and ArrayExpress)

Please outline where this information can be found within the submission (e.g., page numbers or figure legends), or explain why this information doesn’t apply to your submission:

Yeast two-hybrid (Figure 2A, Figure 2-figure supplement 1A), Co-IP (Figure 2B, Figure 2-figure supplement 1B and C), *in vivo* pull-down (Figure 2D, Figure 6A), Firefly luciferase complementation imaging assay (Figure 2C), Gel filtration assay (Figure 2-figure supplement 7), enzyme activity assay (Figure 2G, Figure 2- figure supplement 2 and 3), MST assay (Figure 2-figure supplement 5), SAM, ACC and ethylene measurement (Figure 3, 6B-D and Figure 3-figure supplement 2), Northern and western blot and infection rates (Figure 1B-D, 4B-D, 5B-D, 6G-I, Figure 2-figure supplement 1E, Figure 3-figure supplement B and C, Figure 4-figure supplement 1B-D, Figure 5-figure supplement 2B-D, ) were obtained from three independent biological replicates.

qRT-PCR for gene expression assays (Figure 6E, Figure 2-figure supplement 1D, Figure 3-figure supplement 1A, and Figure 5-figure supplement 3) were obtained from three independent technical replication and biological replicates.

For all the experiments we always chose the plants in the same growth status to analysis infection rates, genes expressions, enzyme activities and etc. Other plants will be excluded.

**Statistical reporting**

- Statistical analysis methods should be described and justified
- Raw data should be presented in figures whenever informative to do so (typically when N per group is less than 10)
- For each experiment, you should identify the statistical tests used, exact values of N, definitions of center, methods of multiple test correction, and dispersion and precision measures (e.g., mean, median, SD, SEM, confidence intervals; and, for the major substantive results, a measure of effect size (e.g., Pearson's r, Cohen's d)
- Report exact p-values wherever possible alongside the summary statistics and 95% confidence intervals. These should be reported for all key questions and not only when the p-value is less than 0.05.

30 rice plants used for virus-infection assay each and analysis of infection rate, see Figure 1D, 4D, 5D, 6I, Figure 4-figure supplement 1D and Figure 5-figure supplement 2D (details see Supplementary file 2). Student’s *t*-test was performed for comparison. Error bars indicate SD, **P*<0.05, ***P*<0.01.

n=6 rice plants from each rice line for SAM, ACC and ethylene measurements, see Figure 3, Figure 3-figure supplement 2, Figure 6B-D and materials and methods. Tukey’s honestly significant difference post hoc test was performed for multiple comparison. Error bars indicate SD, P<0.05.

n=100 from 8 rice plants for analysis of seed length and width, see Figure 1-figure supplement 1C and D. Tukey’s honestly significant difference post hoc test was performed for multiple comparison. Error bars indicate SD, P<0.05.

qRT-PCR for gene expression assays from three independent technical replication and biological replicates, see Figure 6E, Figure 2-figure supplement 1D, Figure 3-figure supplement 1A, and Figure 5-figure supplement 3. Student’s *t*-test was performed for comparison. Error bars indicate SD, **P*<0.05, ***P*<0.01.

Please outline where this information can be found within the submission (e.g., page numbers or figure legends), or explain why this information doesn’t apply to your submission:

(For large datasets, or papers with a very large number of statistical tests, you may upload a single table file with tests, Ns, etc., with reference to page numbers in the manuscript.)

**Additional data files (“source data”)**

- We encourage you to upload relevant additional data files, such as numerical data that are represented as a graph in a figure, or as a summary table
- Where provided, these should be in the most useful format, and they can be uploaded as “Source data” files linked to a main figure or table
- Include model definition files including the full list of parameters used
- Include code used for data analysis (e.g., R, MatLab)
- Avoid stating that data files are “available upon request”

Please indicate the figures or tables for which source data files have been provided:

N/A
